# Extracellular vesicles derived from mesenchymal stem cells as a potential therapeutic agent in acute kidney injury (AKI) in felines: review and perspectives

**DOI:** 10.1186/s13287-021-02573-6

**Published:** 2021-09-15

**Authors:** Magdalena M. Kraińska, Natalia Pietrzkowska, Eliza Turlej, Li Zongjin, Krzysztof Marycz

**Affiliations:** 1 International Institute of Translational Medicine (MIMT), Jesionowa St 11, 55-114 Malin, Wisznia Mała, Poland; 2 Department of General and Transplant Surgery, Warsaw Medical University, Nowogrodzka St 59, 02-014 Warszawa, Poland; 3 Department of Experimental Biology, Wroclaw University of Environmental and Life Science, Norwida St 27B, 50-375 Wrocław, Poland; 4 Nankai University School of Medicine, 94 Weijin Road, Tianjin, 300071 China

**Keywords:** Acute kidney injury, Cats, Mesenchymal stem cells, Extracellular vesicles, Apoptosis, Inflammation, Oxidative stress, Fibrosis

## Abstract

Mesenchymal stem cells (MSCs), known from their key role in the regeneration process of tissues, and their abilities to release bioactive factors like extracellular vesicles (EVs) could be considered as a potential, modern tool in the treatment of AKI (acute kidney injury) in both human and veterinary patients. The complex pathophysiology of a renal function disorder (AKI) makes difficult to find a universal therapy, but the treatment strategy is based on MSCs and derived from them, EVs seem to solve this problem. Due to their small size, the ability of the cargo transport, the ease of crossing the barriers and the lack of the ability to proliferate and differentiate, EVs seem to have a significant impact on the development such therapy. Their additional impact associated with their ability to modulate immune response and inflammation process, their strong anti-fibrotic and anti-apoptotic effects and the relation with the releasing of the reactive oxygen species (ROS), that pivotal role in the AKI development is undoubtedly, limits the progress of AKI. Moreover, the availability of EVs from different sources encourages to extend research with using EVs from MSCs in AKI treatment in felines; in that, the possibilities of kidney injuries treatment are still limited to the classical therapies burdened with dangerous side effects. In this review, we underline the significance of the processes, in whose EVs are included during the AKI in order to show the potential benefits of EVs-MSCs-based therapies against AKI in felines.

## Treatment strategy in AKI in cats: current treatment regimens

The main aim of the AKI treatment is preventing from renal damaging by treating underlying cause and supporting additional possibilities in case to minimize the clinical sequelae of uremia.

Despite extensive research using rodents model, that partially allow to determine the factors involved in the pathophysiology of AKI, the differences related from divergence of innate and adaptive immune response between human and rodents made impossible to directly transfer therapeutic scheme from animal to human clinical practice.

The application of the large animal models (LAMs) included cats, probably will make more attractive the studies of the sequence of events, that occur in the kidneys of both humans and animals with ischemic AKI and allow to establish the proper scheme of treatment [1].

The term AKI (acute kidney injury), originally introduced in the human nephrology, replaced the term “acute kidney failure” (ARF) in the human and veterinary nephrology due to its precision in kidney status evaluation and greater stratification of cases with regard to severity and prognosis. The introduction of this term allows to recognize the potential for earlier treatment and recovery of kidney disorders, while the ARF term refers only for patients requiring renal replacement therapy (RRT) [2].

Acute kidney injury (AKI) is a serious, rapid and complex disorder of renal function associated with accelerated chronic kidney disease (CKD) in both, human and animals. Nearly 8–16% of all hospitalized patients with acute illness suffer from AKI, that is diagnosed by the presence of albuminuria or by the enhance of glomerular filtration rate (GFR) [3].

The key diagnostic criteria of AKI included an increase in serum creatinine (> 0.3 mg/dl) within 48 h, or more than 1.5 times according to the baseline results, that is known or presumed to have occurred within the prior 7 days. It could be diagnosed also, when the urine volume is less than 0.5 ml/kg/h for 6 h [4].

Common known risk factors of AKI are drugs/toxins, sepsis and also the IR (ischemia/reperfusion), that finally lead to decrease in GFR and to the tubular cell death.

In general, according to the etiology, AKI could be divided into the three types; prerenal, renal and postrenal, where the prerenal is associated with the azotemia, and is well characterized as a decrease in GFR due to disorder of the renal pressure perfusion totally without any changes in renal parenchyma. In normal conditions, kidneys concentrate the urine maximally and directly reabsorb sodium due to an increase in intravascular volume that finally leads to the normalization of renal perfusion. The disturbances in this process lead to AKI development.

Postrenal causes of AKI are mainly defined by acute obstruction to urinary flow, due to enhanced intratubular pressure. Moreover, acute urinary tract disorders could induce the impairment of renal blood flow and inflammatory processes that could contribute to the decrease in GFR. Such obstructions in the urinary tract are known as a common causes of the AKI development and are typically represented as anuria, nocturia or intermittent urine flow (e.g., polyuria).

From the other side, the renal etiologies of AKI can be recognized related to the damaging of the tubules, glomeruli, interstitium or intrarenal blood vessels [5].

It is worth noting that ischemia–reperfusion, known as common AKI pathological cause, ends with organs regain perfusion in response to the oxidative stress and inflammation process as a consequence of the activation of the series of cytokines/interleukins (IL) and tumor necrosis factor (TNFα) that are involved in the promotion of the oxidative stress and apoptotic processes leading to the renal dysfunction [6].

Cats and dogs are also affected by the AKI in similar way to human. Regardless of AKI etiologies and treatment type, the mortality rates in cats associated with AKI are equaled to 53% and the greater mortality is observed in those with the non-infectious etiology.

Among the most important factors contributing to the AKI development in felines are nephrotoxic substances involving lily plants, non-steroidal anti-inflammatory drugs (NSAIDs), polyethylene glycol (PEG), aminoglycosides, doxorubicin, vitamin D analogs (VDA) and besides melamine and cyanuric acid detected in food, in a lesser extent ischemic injuries, sepsis and infection involving upper urinary tract, known as pyelonephritis and neoplasia, or prolonged urine tract obstructions [7].

Typical symptoms of AKI in feline are the lack of appetite that dominates together with mild gastric symptoms like vomiting, diarrhea, polydipsia, anorexia, fetor *ex ore* and general dehydration [7].

The diagnosis of AKI should include always hematological (anemia and/or neutrophilia) and biochemical results (the evaluation of azotemia severity) as well as the results of liver activity tests [8].

Additionally, tests allowing to determine the presence of metabolic acidosis, which accompanies AKI, such as bicarbonate production in the kidneys, ability to excrete hydrogen ions, uremic acids levels, feline pancreatic lipase immunoreactivity determination and urine specific gravity measurements should be included in the diagnosis strategy [8].

The new grading system for AKI in cats and dogs, proposed recently by IRIS (International Renal Interest Society), which is based on the creatinine concentration and urine output, is used for diagnosis, management and prognosis prediction. This system is particularly important in cats with unstable kidney disease and allows to define cats with moderate or severe AKI (grade III, IV and V) with progressive degree of renal parenchyma disorder or functional damaged (uremia).

Now, each AKI grade is sub-graded, based on whether the patients are: oligoanuric (O; oliguria) non-oliguric (NO > 1 ml/kg/hr) or require RRT (renal replacement therapy). The assessment of urine production is very important in the sub-grading of AKI, due to its significance in therapy and final outcomes (e.g., allows to choose the optimal status corresponding to the extension phase of AKI for treating patients with cell or extracellular vesicles-derived therapies) [8].

It is also worth to note that there were identified renal biomarkers that may be useful as the indicators of early kidney damaging prior to development of azotaemia, that could be measured in urine and/or blood and involving neutrophil gelatinase-associated lipocalin, kidney injury molecule-1, gamma-glutamyl transpeptidase, N-acetyl-β-glucosaminidase, β-microglobulins, retinal binding protein and cystatin C [8].

AKI, known from its multifactorial and pathophysiology complexity, could be divided into four basic stages that are involved in the idea of the advancement of the process. From the point of view of therapy, the crucial stage is extension phase that occurs immediately after initiation stage [7].

In the early phases like the initiation phase, when the renal tubular epithelial cell injury is detected related to spatially and temporally disruption of the normal filamentous cytoskeleton in the cell, the therapeutic intervention could have weaker effect [5].

In turn, in extension stage presented symptoms correlate with severity of the kidney injuries, despite that, this stage lasts only till 48 h, so the “window time” is very limited and difficult to establish.

An extension stage presents with ongoing hypoxia starting after the initial ischemic and extensive inflammatory response. Both of these processes are pronounced in the corticomedullary junction (CMJ) or in outer medullary renal region.

The key role, in this stage, plays a damage of vascular endothelium due to enhanced vascular reactivity for vasoconstrictive agents and decreased for vasodilating. The observed disruption of the endothelial cells contributes to enhance the expression of ICAM-1 (intercellular adhesion molecule-1) and P- and E-selectins involved in the interactions between endothelial cells and leukocytes and finally leads to inflammation process development. It could be recognized as severely reduced blood flow, stasis and accumulation of red and white blood cells and by the epithelial ramifications.

In detail, during this phase, cells continue to undergo the injury and death (necrosis or apoptosis) predominantly, in the outer medulla. In contrast, the proximal tubule cells in the outer cortex, where blood flow has returned to the normal levels, actually undergo cellular repair and improve morphologically. Moreover, during this stage, a cellular injury continues in the CMJ region and the GFR falling down stimulates release of cytokines and chemokines and further enhances the inflammatory cascade. It is supposed that interrupting the amplification of this inflammatory cascade may have therapeutic implications [5].

Further stages, called maintenance characterized by the decrease level of creatinine and releasing symptoms typical for uremia in patient with hypertension, fluid imbalance and hyperphosphatemia, can last up to two weeks and finally recovery stage characterized by GFR rate recuperation and polyuria, which can last for months, has a little of importance in case of AKI treatment [9].

Taking into account the complexity of the pathomechanism of AKI in feline conservative management including fluid therapy (fluids imbalance improvement) in the course of anuria/oliguria, the correction of electrolyte and acid–base imbalance with avoidance of nephrotoxic medications (e.g., nephrotoxic antibiotics like gentamicin, NLPZ) as well as nutritional support is considered as a first-line therapy [7].

Each of previously mentioned therapeutic strategy has its own limitations. For example, fluid volume status in cats with AKI should be checked firstly, due to the risk of dehydrated/hypovolemic or fluid volume overload. The clearly visible dehydration in cats needs to be recovered by fluid administration that corrects the electrolytes imbalance and acid–base disorders and replaces the fluid volume, although the excessive fluid administration leads to reduce renal blood flow (RBF) and decrease GFR and urine secretion. This situation is particularly visible in anuric and oliguric cats, and entails the decrease in creatinine concentrations in serum/plasma. Nonetheless, fluid overload with concurrent anuria/oliguria is the indication for dialysis [10].

In cats, a good solution for delivering accurate fluid volumes is drip pumps and syringe drivers filled with compound sodium lactate (Hartmann’s lactated Ringer’s) and physiological saline in case of accompanying hypochloremia, hyponatremia or severe secondary hyperkalemia. It is recommended, in such cases, to treat the cats with a fluid bolus of 10–15 ml/kg over 10–20 min, until the cat becomes hemodynamically stable. During all this process, the fluidics loosed by urine output, vomiting or diarrhea should be monitored [11].

This monitoring is very important, in particular in response to the fluid therapy, and could be achieved by placement of a urinary catheter combined with a closed collection system. Both of them should be placed, when the cat is stabilized under a short-time general anesthetic that induces appropriate muscle relaxation. Once the normal urine output is achieved, the urinary catheter should be removed as its presence will increase the risk of urinary tract infections.

Similarly, if a urinary tract obstruction or rupture is identified then placement of a urinary catheter or rarely surgical intervention may need to be considered [8].

The main disadvantage of fluid therapy is a risk of electrolytes loosing. Hyperkalemia, probably the most common complication of AKI, is a result of decreased renal excretion of K^+^ ions, and in the severe form can lead to the bradykardia [10, 11].

Other way of AKI treatment is diuretics that are used in oligoanuric veterinary patients despite fluid therapy. Furosemide and mannitol are the most commonly used, although there are no clinical outcomes, confirming that diuretics can improve status in advanced AKI. Despite that, the application of diuretics in veterinary plays a crucial role in volume management and the loop diuretics predominate osmotic in AKI patients due to their high efficacy and margins of safety.

To the most dangerous, potential adverse effects associated with the mannitol administration that belong to fluidics overload due to high osmolality and the development of a renal tubular morphologic lesions, so-called *osmotic nephrosis*, characterized by swelling and vacuolization of the RTCs. Although the effect of alone administered mannitol in cats with AKI has not been elucidated, a combined therapy involving mannitol application and fluid therapy in healthy, awake cats seems to not affect on GFR ratio or urine output as compared to the fluid therapy alone [10, 11].

At the same time, it is suggested that the ability to respond on diuretics could be a marker of less severe renal injuries associated with better prognosis.

From the other hand, in patients with renal failure a RRT should be considered, although it is indicated only for those cats that are expected to regain kidney function. The intermittent hemodialysis and continuous RRT are used mainly for acid–base, electrolyte or fluid disturbances, as well as in the cases of uremia in AKI.

The main problems in this case seem to be a size of the extracorporeal circuit and associated with this volume of blood; the chemical composition of the fiber membranes used for hemodialysis and ultrafiltration of plasma is required to obtain a neutral fluid balance. For that reason, it is not allowed to perform RRT in cats in institutions that have no access to plentiful donor RBC suppliers. Despite that, the RRT therapy is labor intensive and associated with many additional complications that limited its usefulness in cats with AKI [10, 11].

Among others, AKI treatment strategies and anti-emetic therapy with 5-HT3 antagonists or neurokinin-1 are taken into consideration. Except that, also anti-secretory drugs like proton pump inhibitors could be considered during the prevention of stress-associated mucosal bleeding that often accompanies to AKI, despite that, even short-term administration of them can result in toxic accumulation of aluminum in the organs.

Another possibility is to use a dopamine as a pharmacologic agent in AKI to enhance renal blood flow. Despite the putative dopamine receptor (DA-1) identification in the feline renal cortex, it seems that the increase in renal blood flow in cats is associated rather with systemic cardiovascular effects than by dopamine infusion.

In the analog of DA-1, fenoldopam has higher affinity to DA-1 receptors in cats kidneys but cannot be used by long term due to increase in creatinine clearance and urine output in healthy cats, and is not recommended as a first-line drug due to its cost, undetermined efficacy and the pharmacokinetic properties in felines with AKI [10].

All these problems limit the usefulness of actual known AKI therapies and are a main cause of searching a new, more safety scheme of AKI treatments in felines [7].

## The modern ideas in AKI treatment in cats

### The role of MSCs therapies

Stem cell therapy is an innovative field of scientific investigation with tremendous potential for clinical application that holds a big promise for the treatment of a variety of diseases also in the veterinary medicine. Based on the knowledge of desirable properties of mesenchymal stem cells (MSCs), the therapy has potential for treatment of both acute and chronic kidney diseases in cats.

MSCs could be obtained from different sources, among others bone marrow, adipose tissue, peripheral blood, the lungs or the heart; however, the minimal criteria that allow human MSC defining included positive expression of CD105 (SH2), CD73 (SH3), CD44 and CD90, while negative for CD45, CD34, CD14, CD11b, CD79α, CD19 and HLA-DR. Typically, they are characterized by the ability to adhere to the plastic under standard cell culture conditions and ability to differentiate into osteoblasts, chondroblasts or adipocytes under appropriate conditions [12].

MSCs are thought to assist in tissue regeneration and repair through a multitude of mechanisms that include cell to cell contact, the secretion of mediators and extracellular vesicles (EVs), as well as by the formation of membrane nanotubes (TnTs), that transfer the trophic factors (including mRNAa and miRNAs) and mitochondria between cells in case to repair damaged organs.

MSCs are known from the secretion of many bioactive factors that influence on the angiogenesis and inflammation processes. Moreover, the factors released from MSCs are included in the immune system regulation and can impact on the stem cells recruitment to the local organ injuries. Their role in the stimulation of the stem cell action is associated with their abilities to stimulate stem cell survival, proliferation and differentiation [13].

Despite the species, MSCs could interact with the immune cells like T and B cells, natural killers (NK), monocytes/macrophages and dendritic cells (DCs) and even with neutrophils. In case of felines, it was discovered that MSCs share the ability to mimic the immunomodulatory phenotype between human, equines and canines MSCs. However, the common difference between them is their ability to inhibit activation of T cells proliferation and reduction of the reactive oxygen species (ROS) secretion by neutrophils [14].

Interestingly, the role of the systemically administered MSCs in the AKI treatment is mainly focused on the ability of the MSCs to inflammation modulation. For this purpose, the autologous stem cells or the allogeneic stem cells harvested from recipient could be used. In rodent models, both MSCs sources effect positively on preservation of the kidney architecture, improvement of renal function and on anti-inflammatory and anti-oxidative effects [15].

The therapeutic efficacy of the MSCs was observed in reducing both, AKD and CKD in animal model; therefore, rapidly the further studies on safety and efficacy of allogenic MSCs infusion were performed. Their application represents, undoubtedly, the optimal option in AKI treatment due to their physiological role and ability to improve the renal function that is associated with GFR ratio increasing, alleviation of oxidative stress-induced cell senescence and increase in proliferation of the kidney cells during IRI-model [16].

Using these cells in AKI treatment required to define the best route of the administration, the number of cells per administration, the number of the injections, the understanding of the interactions between MSCs and the other cells and finally identifying side effects of MSCs are observed. It seems that the intravenous injection, the multiple injections, cell size and the total number of cells could increase the chance of pulmonary entrapment after MSCs administration [17].

Moreover on the MSCs properties and usefulness during AKI treatment impact, two factors; first—genetic modifications can strengthen the paracrine effect, homing, immunomodulatory, anti-inflammatory or tissue repair, and the second—preconditioning performed by the culturing MSCs in a hypoxic environment enhances their multipotency and migratory/proliferative potential, which in turn inhibits the MSCs differentiation into tumor-associated fibroblasts in in vitro conditions and tumors growth in in vivo conditions [18].

The beneficial effects of MSCs application in experimental models of AKI were associated with the further protection against AKI development and faster renal repairing in AKI induced by I/R.

It is worth to note that MSCs only transiently traffic in the post-ischemic kidney declining within 1 day with the majority of the cells homing in the lung and spleen. Surprisingly, MSCs that produce large amounts of the VEGF and IGF-1 were repressed by siRNA that impair their role in the recovery from AKI.

In turn, after glycerol-induced injuries, MSCs homing was suggested to be associated with the CD44 expression and its interaction with the hyaluronic acid as an inhibitor of CD44 expression on the surface of transplanted cells results in the decrease in cells homing and repairing.

Mostly, the preclinical studies proved that the administration of MSCs systematically provides the long-term support by either directly replenishing damaged tissue or by interacting with surrounding cells to promote endogenous repairing [19].

Moreover, the effective transplantation of MSCs assumed that nearly 100% of the cells remain active after infusion into the subject and the units of the activity of the transplant would approach steady state in the timescale of days to weeks after administration, although normalization of the cell concentration on the plot takes place during 1–120 h, while the kinetics of MSCs grafting is much more transient than expected half-life (24 h) [19].

It should be noticed that after arbitrarily choosing a minimum effective concentration of MSC for therapy and transposing it from the timescale to a biological response, the duration of transplanted MSCs and its therapeutical effect was extremely short (less than 24 h), precisely corresponding with the measured serum cytokine levels. Ultimately, the solution that seems to be administered by the successive doses of MSCs within a shorter treatment period for sure allows for the maintenance of MSC therapy within a therapeutic window and sustains a long-term biological response [20].

The explanation of the feline MSCs response to the inflammatory stimuli is associated with the discovery, that incubation of MSCs with allogeneic peripheral blood mononuclear cells (PBMCs) stimulated with lectins (concanavalin A—ConA, interferon gamma (IFNγ) TNFα) enhances expression of genes related to the immunomodulatory mediators including indoleamine 2,3-dioxygenase (IDO)1, programmed death ligand-1 (PD-L1), interleukin (IL)-6, cyclooxygenase 2 (COX2) and hepatocyte growth factor (HGF) [14].

In the same time, it was noticed that feline’s aMSCs (adipocyte-derived mesenchymal stem cells) are able to secrete IDO, prostaglandin E2 (PGE2), IL-6, vascular endothelial growth factor (VEGF), IL-8 and transform growth factor beta (TGFβ), contributing to inhibition of release in the proinflammatory cytokine TNFα and lymphocyte proliferation in the presence of high concentrations of IFNγ in the in vitro conditions [21].

Research helped to define the transcriptome of the three feline’s aMSC-derived cell lines using apoptosis, cell adhesion, response to oxidative stress and cell differentiation evaluation; however, feline’s models of AKI seem to be ideal for cats, dogs and also humans studies on AKI treatment using MSCs.

Importantly, AKI can be potentially reversible if diagnosed early and treated aggressively, so effective regenerative therapy seems to be a good solution in this case. However, till now, such kinds of therapies in AKI are limited due to the lack of the knowledge about molecular background of the regeneration process. Till now, in the literature there is description only for a few of studies conducted using feline’s MSCs.

First research using feline-derived MSCs was concerned for isolation and characterization of cells isolated from bone marrow (BMSCs) in 2002 and later readily expanded from fat, fetal fluids and amniotic membranes. Due to ease of attainment and their superior proliferative abilities, the adipose-derived MSCs are now most commonly used in the clinical applications. Besides, the feline MSCs are not well categorized and known, and ongoing research confirms that they have similar immunomodulatory potency as more recently defined human MSCs [22].

There were a few pilot studies with MSCs administration in case of the treatment of cats with CKD and AKI in the ischemic kidney model. Investigation, that MSCs could be administered in AKI model treatment, showed no difference in AKI development or urine specific gravity, proteinuria, GFR and histopathology in cats. At this time, MSC therapy for feline CKD and AKI ischemic model should be considered an experimental and unproven therapy [22].

The role of using the MSCs for AKI treatment is considered based on their protective role and commitment in the oxidative stress suppression. The observation in rats model of adriamycin-induced nephropathy MSCs, may reduce the oxidative stress and inflammation and could act as a potential neuroprotective factor for hippocampal neurons against synapses damages, contributing to the further development of studies on MSCs application in the AKI treatment. It was established that MSCs transplantation could minimalize glomerulonephritis through antioxidation and anti-apoptosis effects in nephritic rats [23, 24].

It is not without significance that the intravenous applications of MSCs have no life-threatening side effects. Clinical signs follow its administration including only vomiting, nausea and mild disturbances in the respiratory rate [25].

In the literature, a description of the case of febrile (104.4°F), lack of appetite, exhibited open-mouthed breathing and ptyalism about 1 h after intravenous cell administration in human. However, all of the resolved symptoms, beside the lack of appetite, disappears within a few days, and would last only for 24 h after subcutaneous administration of fluids and primary care.

A single intravenous (IV) administration of allogenic MSCs appears to be safe and induces only mild reactions, previously reporting a hyperthermia as a secondary reaction of immune system to the cells [25].

The main problem with the MSC administration to the cats is obtaining high amounts of the cells, what causes that the injection of the MSC should be divided into a few parts. It was noticed that better results are obtained after using aMSCs from cryopreserved adipose tissue than from cryopreserved MSCs.

There is no recommendation to use directly cryopreserved aMSCs due to its involvement in the treatment-related side effects. The most likely explanation of this is an instant blood-mediated inflammatory reaction, which results in clumping of the cells due to their contact with the blood and the subsequent potential for micropulmonary thromboembolism [26].

In summary, it is believed that although MSC therapy has the potential for the great applicability to feline diseases, there are still additional questions; those should be answered like logistics of its use, the optimal route of their administration and the ideal source of the MSCs (allogeneic vs autologous, culture-expanded vs SVF) as well as the impact of the tissue donor status (age, disease status, sex) on their function [26].

Is not well known, if the way and the speed of administration of MSCs can influence on the side-effect presentation or the allergic reaction in patients. It can be said that adverse effects are barely significant after MSCs administrating encourages to further develop therapy using MSCs or derived from the EVs.

### The role of extracellular vesicles in AKI treatment in cats

EVs are involved in the mechanism of the intracellular communication between donor and recipient cells, and based on the typical for them cargo, size range and cellular origin could be divided into microvesicles (MVs), exosomes, ectosomes, shedding vesicles and microparticles (MPs) among others shedding by healthy cells [27].

It is well known that EVs could be secreted by nearly all cells, healthy or apoptotic and could carry different cargos including proteins, lipids and various kinds of RNA (mRNA, miRNA, lncRNA and many others) and DNA (mtDNA, ssDNA or dsDNA) [23].

Interestingly, some proteins in EVs are shared between specific types of them and therefore might be used as a typical for their markers, while the content of EVs depends on the lineage of the parent cell and its detection could improve the ability to recognize their origin in the tissues or organs. Moreover, within the same cell lineage, the composition of released EVs can be differed depending on the activation state of cells, viability or even ROS releasing [23].

The smallest EVs, called exosomes, range between 30 and 150 nm in diameter. They are generated within the cellular endocytic compartment through reverse budding of the limiting membrane of MVBs (multivesicular bodies) as a ILVs (intraluminal vesicles). Further, the MVBs could either fuse with lysosomes, what leads to ILVs degradation or with plasma membrane, what leads to ILVs releasing to the extracellular environment and body fluids.

Generally, MVs are larger than exosomes and are generated by shedding the plasma membrane. The route of their formation is not well established; however it is known that the cytoskeleton components like actin and microtubules, together with molecular motor proteins (kinesins and myosins) and SNAREs (SNAP REceptor) machinery, are required to their releasing [28].

The third type called apoptotic bodies (ApoBDs) is released during the apoptosis, which involve many cellular signaling pathways depended on a wide range of stimuli, intracellular proteolytic cascades or DNA cleavage. In opposite, to the different types of EVs, ApoBDs contain organelles derived from origin cells.

It is interesting that EVs could participate in the maintenance of both physiological and pathological conditions involving cancers, neurodegenerative, rheumatic and even infectious diseases [23]. Moreover, EVs may have beneficial effects in the regenerative medicine due to its release by stem cells and their abilities to mimic their function [29].

According to the literature, EVs could be recognized in the kidneys and participate in their pathophysiology by mediating intercellular communication, transferring their contents and activating signaling pathways in the target cells.

The urinary EVs are actively released by almost all renal cells along the nephron and the urogenital tract as well as by infiltrating inflammatory cells. Moreover, EVs can be uptaken using lipid drafts along the urogenital tract and can affect the function of recipient cells.

The glomerular filtration protects renal nephron from EVs presence in the blood entering into their lumen. Secreted into the extracellular fluids, EVs could play an important role in the renal signaling solely by stimulating different cell types and by cooperating with the vascular compartment and various immune cells [30].

Moreover in the healthy conditions, nephron cells continuously release EVs containing nearly identical cell surface proteins to those from their origin that could fuse with the target cells and after biological molecules releasing induce cellular response in the target cells.

EVs can play a key role as a messenger in the nephrons and specifically interact with the recipient cells that use cilia to uptake the EVs. Based on the electron microscopy results, it was established that EVs adhere to the cilia and emerge from an intracellular vesicle near the base of the cilia*.* The process of EVs uptaking is typically concentration dependent and could be increased in the cellular stress situation [31].

A common interest in EVs studies is associated with their ability to act as a biomarkers of disease, e.g., EVs isolated from urine can reflect the development of AKI [17]. However, their therapeutic potential in many diseases also cannot be overlooked. In major perspectives, the promising aspects of using EVs in therapeutic schemes are associated with their abilities to transport genetic information, proteins, lipids, etc., between cells and with the stability of their cargo in in vivo conditions [32].

The important issue that should be taken into account, during consideration of EVs application in clinics, is its biodistribution in the body and half-life. According to the literature, the general half-life of the EVs in in vivo conditions determined in athymic nude mice was approximately 30 min, and the total time required to clear the blood after intravenous injection of EVs was approximately 6 h [33].

The analysis of EXOs, that are the best studied EVs in the context of clinical usage, showed that EXOs had short half-life in the blood circulation (about 2 min after intravenous injection to mice) that could significantly affect on the success of therapy with their implementation.

The biodistribution of exogenously administered EXOs has been best studied using lipophilic fluorescent dyes such as lipophilic near-infrared dyes; DiD (1,1’-dioctadecyl-3,3,3′3’-tetramethylindodicarbocyanine perchlorate) or DiR (1,1’-dioctadecyltetramethyl indotricarbocyanine iodide) after intravenous injection of fluorescent-labeled EXOs released from MSCs to healthy mice. The results of these experiments revealed that EXOs accumulate mainly in the spleen and in the liver; however in case of AKI mice model, EXOs could also accumulate in kidneys.

Further experiments using HEK293T cell line (human embryonic kidney) proved that there is a link between the route of EXOs administration and the site of their accumulation. It was observed that EXOs administered intravenously accumulate mainly in the liver, while those administered intraperitoneal or subcutaneous liver, pancreas or gastrointestinal tract. Similarly, the biodistribution of the EXOs obtained from the body fluids (from bovine milk) administered orally accumulates the liver, lungs, kidneys, pancreas, spleen, ovaries and brain within 4 days, while administered intravenously predominantly the liver [34].

### The application of EVs-derived from MSCs in the treatment of feline AKI

Interest in EVs application in the treatment of AKI using MSCs was associated with recent discoveries those proved that MSCs may promote recovery from AKI, mainly in the paracrine mechanism based on the mediators released from them [25].

In comparison with stem cells, EVs are characterized by higher physicochemical stability and stronger signaling [35, 36]. In addition, they have a lower ability to induce an immune response due to their low immunogenicity, that in case of treatment with using EVs allow on using particles from different stem cell sources [37]. This approach, in case of tissue repair, unlike cell therapy, is associated with a lower risk of complications especially graft versus host disease (GvHD) or cytokine release syndromes [37].

Moreover, EVs are characterized by high biocompatibility, and due to their small size, can easily overcome biological barriers [38, 39] and achieve target tissues in a very short [28, 36, 38, 39].

EVs, unlike stem cells, do not have the ability to proliferate or differentiate, which reduces the likelihood of the formation of abnormally differentiated cells or neoplasms [36, 40].

It is worth to note that EVs released from MSCs express proteins involved in the MSCs self-renewal and differentiation and the nucleic acid composition (mRNA and microRNA) typical for MSCs.

Taking all these features into consideration, it is not surprised that EVs isolated from MSCs become so fast a potential agent in treatment of kidney injuries. As far as their source, in the initial studies, researchers used EVs obtained from bone marrow MSCs (BM-MSCs) and observed the recovery of the injured renal tubular cells, cell proliferation and apoptosis inhibition during the AKI induced by glycerol injection model, and that encouraged them to conduct further studies, using several models of AKI and CKD [22, 41].

This turns out that the treatment strategy based on EVs isolated from stem cells may be as effective or even more effective in AKI than classical cell therapy [42]. For these reasons, MSC-derived EVs seem to be a promising therapeutic agent in AKI.

The beneficial effects of EVs-derived from MSCs, during the course of AKI, are associated with their anti-apoptotic, anti-fibrotic and immunomodulatory abilities.

The problem is that, according to the literature obtaining large amounts of EVs required for the clinical application should be a big problem (the literature data show that cells secrete only about 100 pg/10^6^ cells/day of therapeutic mediator that corresponds to 0.1 fg/cell/day). Based on this, nearly 100 × 10^6^ cells should be given intravenously to obtain 10 ng of a therapeutic molecules, what is difficult to achieve; however, the positive sides of such therapy for sure rewards efforts taking for EVs massive production [5].

#### The anti-apoptotic effect of EVs during AKI treatment

A common process, during the AKI course, is the apoptosis of renal tubular epithelial cells (RTECs), that in mostly cases is a consequence of the exposure to toxic agents.

RTECs, normally involved in the absorption of glucose, amino acids, etc., from the primary urine, constitute an outer layer of cells in the renal tubule [43]. Classically, they are characterized by the expression of the death cell receptors belonging to the tumor necrosis factor (TNF) superfamily, strictly contributing to the apoptosis induction.

RTECs disturbances during the pathological conditions are related to the AKI etiologies, e.g., in ischemia/reperfusion AKI, RTECs loss the brush border and cell polarization, leading to the tubular obstruction, necrosis and apoptosis. In opposite, in AKI induced by cisplatin, damaging of DNA and mitochondria induces the inflammation and apoptosis. However, the most serious consequences are associated with the AKI induced by sepsis due to its impact on the renal vasodilation caused by iNOS (inducible nitric oxide synthase) releasing [44].

Recently performed studies have demonstrated that MSCs application during the course of AKI could protect RTECs against apoptosis, due to their role in the acceleration of the endothelial cells proliferation and angiogenesis promotion. This observation strictly implicates further studies using MSCs and derived from them EVs in the range of apoptosis inhibition [45].

In the histopathological studies, comparing the effect of human MSCs and MVs derived by MSCs injected intravenously to SCID mice suffered from AKI induced by glycerol observed a visible reduction in the casts of vitreous tubules, cytoplasmic vacuolation and necrosis of RTECs. In addition, mice treated with MVs present reduced, blood urea nitrogen (BUN) and creatinine (Cr) levels [46].

Interestingly, the effect of MVs on RTECs was modified in in vitro conditions and the final reports showed that the renal tubular epithelial cells incubated in the presence of MVs represent limited apoptotic profile. Similar observation related to beneficial effects of MVs derived from human MSCs was made in the glycerol-induced AKI model in mice [46].

A congruous effect of using MSCs-EVs in the same AKI model was also confirmed by Bruno et al. in 2017 in next studies. The pro-regenerative properties of EVs in the same AKI model were observed after a single administration of EVs derived from MSCs, partially due to the presence of mRNAs and miRNAs involved in the modulation of cell division and counteracting with anti-apoptotic pathways including phosphoinositide 3-kinase (PI3K) and the mechanistic target of rapamycin (mTOR) [47].

The efficacy of MSCs-derived MVs was also confirmed in a model of AKI induced by cisplatin. In 2012, Bruno et al. confirmed that MVs exert strong anti-apoptotic effect against RTECs in mice during the course of AKI and showed that repeated administration of MVs released from hMSCs to mice with cisplatin-induced AKI improved kidney status and prolonged the overall survival of mice treated in this way.

Interestingly, multiple infusions of MVs were in this case more effective, than single one, what suggests that the therapeutic effect of MVs was associated with the inhibition of programmed cell death. The salutary effect of these MVs was confirmed in the histological studies, where the tissue morphology appeared normal in the mice treated with several doses of MVs, that was proven by MVs apoptosis counteracting (significant reduction of caspase-1 (Casp-1) expression) [48].

In turn, Zhou et al. in 2013 showed that the administration of EXOs (exosomes) derived from human umbilical cord mesenchymal stem cells (hUC-MSCs) to rats alleviated cisplatin-induced kidney damages, partially by reducing RTECs apoptosis. The protective effect of these EXOs was probably associated with their ability to incorporate into the injured cells followed by acceleration of the recovery of kidneys [17].

Similar abilities of EVs to counteract with apoptosis have been recently confirmed also in the model of AKI induced by ischemic reperfusion injury (IRI) [16, 49].

Gatti et al. in 2011 verified the exerted nephroprotective effect of MVs released by MSCs in rats with AKI caused by ischemia–reperfusion injury by histological observations, that demonstrated that the group, that received MSC-MVs were characterized by significantly reduction of the pathological changes in the renal tubules. It was occurred that directly after injection, MVs accumulate in glomeruli and in injured RTECs stimulates their proliferation. In addition, MVs could act through a mechanism that limits the extension of injury by reducing RTECs apoptosis [50].

A similar study was conducted by Li et al. in 2019, where the authors demonstrated that the injection of EXOs released from MSCs into rats with IRI reduced the number of apoptotic cells, thereby weakening renal dysfunction. MSC-EXOs counteracted apoptosis by reducing the expression of Casp9, Bax (Bcl-2 associated X) and cleaved Casp-3, while increasing the transcription of Bcl-2 (B-cell CLL/lymphoma 2) gene. These results clearly indicate that the protective effect of MSCs-EXOs was due to the inhibition of the mitochondrial pathway of programmed cell death [44].

Additionally, Zhu et al. in 2019 concluded that miR-199a-3p was involved in the anti-apoptotic effect of EXOs secreted by MSCs in the bilateral mouse IRI. This miR was presented in EXOs after inhibiting the expression of semaphorin 3A (Sema3A), that induced the inclusion of pathways, in which protein kinase B (AKT) and extracellular signal-regulated kinase (ERK) were involved leading to the apoptosis limitation [17].

**Fig. 1:**
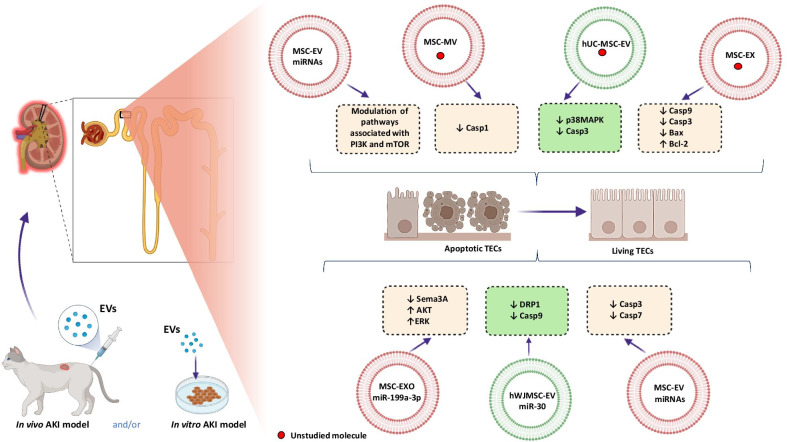
The anti-apoptotic effects of the EVs in in vitro and in vivo conditions

The complexity of the anti-apoptotic effects of EVs is shown in Fig. 1.

Interestingly, EVs released from human stem cells derived from Wharton's jelly (hWJMSCs) also revealed the anti-apoptotic properties. According to Gu et al. studies from 2016, a single administration of hWJMSC-EVs alleviated acute kidney damage in the rat unilateral IRI model in in vivo and in vitro conditions [51].

The pro-regenerative effect of hWJMSC-EVs resulted from the transfer of miR-30, which inhibited that activation of dynamin-related protein 1 (DRP1) was considered as the main modulator of mitochondrial fragmentation, thus reducing their fusion, which consequently counteracted RTECs apoptosis. Moreover, in kidney tissues of subjects treated with hWJMSC-EVs, the content of Casp9 was decreased, which may confirm that the miRNA-30 presented in EVs inhibits mitochondrial pathways of programmed cell death [51].

In addition, the effect of EVs isolated from hBMSCs in the IRI in an in vitro induced model is associated with the reduction of the apoptosis of damaged proximal tubular epithelial cells (PTECs).

It is worth noting that microRNAs could lower the expression of genes involved in the apoptosis process, e.g., Casp-3 as a target for miR-410, let-7a, miR-495 and also for miR548c-5p, while Casp-7 as a target for miR-375, miR-548c-5p and miR-495 [52].

#### The anti-inflammatory effects of EVs released from MSCs in AKI

Independently of the etiology, AKI is an inflammatory disease, where the RTECs damaging initiates an immune response. Generally, it is associated with the presence of the necrotic cells and releasing by them the intracellular molecules called DAMPs (damage-associated molecular patterns) that could activate TLRs (toll-like receptors) on the surface of RTECs, dendritic cells and even on fibroblasts. Moreover, DAMPs are also involved in the recruitment of leukocytes secreting proinflammatory cytokines and chemokines, thus finally leading to extension of kidney injuries [53].

Interestingly, during the AKI course, there are also opposing mechanisms associated with the releasing by lymphocytes and macrophages a wide panel of the anti-inflammatory cytokines.

Macrophages actively participate in the development of the inflammation during the early phases of AKI through releasing TNF-α and IL-6, whereas in the repair stage, they exert anti-inflammatory profile and stimulate RTECs regeneration [53].

The key role of EVs in the immune system is associated with the mediation in both innate and adaptive immune responses, mainly in the context of antigen presentation due to the presence of MHC class I and II molecules on their surface. In turn, the therapeutic effect of the MSCs is observed and derived from them EXOs were observed in IRI. This effect was probably partially associated with the decrease in the expression of pro-inflammatory cytokines; TNF-α and IL-1β, proteins were involved in inflammation processes (NF-κB), macrophage migration inhibitor factor (MAF), plasminogen activator inhibitor 1 (PAI-1) and cyclooxygenase-2 (Cox-2). In addition, a reduced number of CD68 + macrophages in the microscopic analysis seem to confirm this [54].

Moreover, the observations that EXOs derived from mouse bone marrow mesenchymal stem cells (mBM-MSCs) could reduce kidney damages in IRI and improve their function due to expression on their surface the C–C motif chemokine receptors 2 (CCR2), that binds with its ligand CCL2/MCP1 (monocyte chemoattractant protein 1) confirms their role in the regulation of inflammation. MCP1 is known from a strong ability to drive the chemotaxis for among others monocytes. Normally, the expression of CCL2 is inducible and could be noticed in the circulation and in tissues, contributing to leukocytes recruitment and organ repairing [55, 56].

Studies on rats with unilateral IRI demonstrated that intravenous administration of MSC-EXOs alleviates not only strong anti-apoptotic effect, but also tissue injuries that contribute to the improvement of the conditions of the kidneys [44].

It was established that treatment with using MSCs or EXOs derived from MSCs has the ability to decrease the levels of pro-inflammatory cytokine transcripts for interleukin-6, TNF-α and IFN-γ [Li et al. 2019].

In the same time, strong decreased expression of phosphorylated nuclear factor kappa B (p-NF-κB) and a phosphorylated κB inhibitor (p-IκB), together with the increase in IκB expression level ultimately leads to the activation of NF-κB, which by penetrating the nucleus can modulate the expression of many genes involved; among others, those included in the inflammatory and apoptosis processes. This for sure indicates that MSC-EXOs could inhibit the NF-κB pathway, which may be significant during the inflammation development in IRI [44, 57, 58].

Interestingly, it was reported that pulsed focused ultrasound (pFUS) procedure has the ability to enhance MSC therapy in the context of cisplatin-induced AKI. It was demonstrated that pFUS enhances the content of local cytokines thus act as a homing signals for MSCs and increasing their accumulation. From the other hand, the positive effect of pFUS on MSCs application in this model of AKI could be totally independent of increased MSCs homing due to pFUS alleviates inflammation associated with NRLP3 (NLR family pyrin domain containing 3).

NRLP3, together with ASC and pro-caspase 1, states an inflammasome, which is an intracellular protein complex, contributing in the conversion of IL-1β and IL-18 into the mature forms.

**Fig. 2:**
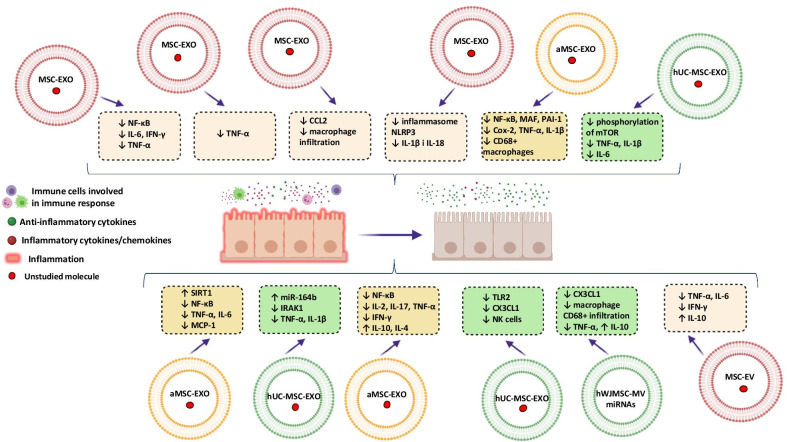
The role of EVs released from MSCs in inflammation and immunomodulation

Importantly, the combined therapy of pFUS and EXOs derived from MSCs decreases the expression of the HSP90 (heat shock proteins), HSP70 and NLRP3 during the AKI induced by cisplatin injection. These HSPs are known from their abilities to regulate inflammation, although their direct effect depends on the context, e.g., knockdown of HSP70 leads to decrease in expression of NRLP3, suggesting its role in the positive regulation of inflammasome function [59] (Fig. 2).

Similar results were obtained in the context of different AKI models; e.g., in gentamycin-induced AKI, the injection of allogeneic MSC-EVs into rats decreased the levels of TNFα, IL-6 and IFNγ, while increasing the level of IL-10 [60].

In turn, in the mouse model of AKI induced by glycerol, the anti-inflammatory effect of EVs released by MSCs was confirmed by the slightly decrease expression of the genes, related to inflammation process [52].

The same effect was observed in the mouse model of AKI induced by sepsis, where treatment with using EXO released by MSCs was contributed into the inhibition of NF-kB pathway, largely activated by the proinflammatory cytokines [61].

Similar observations were reported in the mouse model of cisplatin-induced AKI, treating by EXOs released from MSCs. The application of EXOs in this model alleviates the expression of pro-inflammatory cytokines genes, thus influencing on the level of IL-2, IL-17, TNFα, IFNγ, IL-4 and IL-10 in the serum of mice [62].

In addition, the same effect was observed in case of unilateral IRI treating with EVs-derived from hUC-MSC. The injection of hUC-MSC-EVs into the rats with this model of AKI slightly diminished the kidney damaging due to decrease in a number of NK (natural killer) cells and the expression of TLR2 (toll-like receptor 2) [17].

#### The oxidative stress in the AKI treatment with EVs

Oxidative stress (OS) is a key player during the pathophysiology of AKI. Typically, OS occurs as a result of the excessive accumulation of OS-mediated molecules; ROS (reactive oxygen species) and RNS (reactive nitrogen species). The most important in this group seems to be superoxide anion (O_2_^⋅^), hydroxyl radical (OH), hypochlorous acid (HOCl) and hydrogen peroxide (H_2_O_2_).

Importantly, besides their critical role in the regulation of fundamental cellular mechanisms, their high content could lead to the damaging cellular integrity and capacity. It is worth to underline that ROS could be released by both endogenous and exogenous sources. However, in the context of AKI, a pivotal role plays endogenous sources of ROS production attributed to the mitochondrial electron transport chain, that is strictly associated with the relationship between the numbers of mitochondria and the kidneys susceptibility to damage induced by OS [63, 64].

In general, the accumulation of ROS leads to deficiency and inactivation of NO (nitric oxide) in RTECs during the AKI, thus contributing to the anti-oxidant effect.

OS appearing is usually associated with the Nrf2 (nuclear factor erythroid 2—related factor 2) activation and entering to the cell nucleus, where it attached the anti-oxidant response elements (AREs) leading to their enhanced expression. Nrf2 is considered as a main modulator of the response during the OS that plays a key role in maintaining the redox balance in cells, helping them to survive in adverse conditions [65].

For the first time, the unique abilities of EVs obtained from human Wharton Jelly mesenchymal stromal cells (hWJMSCs), in the context of a reduction of OS, were noticed in rats with AKI treated using MVs. Interestingly, it was demonstrated, during these studies, that even a single application of MVs obtained from hWJMSCs to rats, shortly after the occurrence of IRI (ischemia/reperfusion injury), could reduce the level of ROS, and the same the OS. Probably, the protective role of MVs was associated with the inhibition of NADPH-2 oxidase (NOX2) expression, which is known from its strong pro-oxidative effects [61].

Similar positive effect of the administration of EVs released by hWJMSCs was demonstrated after intravenous administration of EVs to rats with IRI. This procedure lowered OS by increasing the activation of Nrf-2/ARE, contributing to the reduction of tubular damages, and finally improved the kidney function. The reduced levels of MDA (malondialdehyde) and 8-OHdg (8-hydroxy-2″-deoxyguanosine) proved the effectiveness of EVs in alleviating OS.

**Fig. 3:**
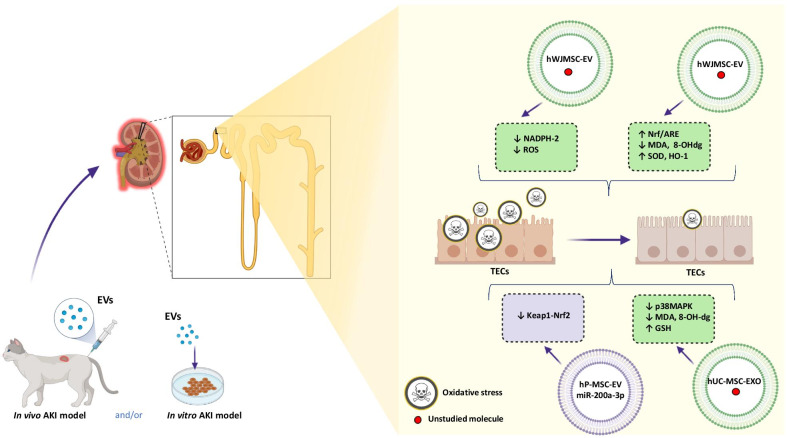
The anti-oxidative effects of the EVs-derived from MSCs

Moreover, increased expression of genes encoding superoxide dismutase (SOD) and HO-1 (heme oxygenase-1) known as enzymes with antioxidant properties, controlled by ARE has been observed, after EVs treating during the AKI [66] (Fig. 3).

#### The anti-fibrotic effect of EVs derived from MSCs during AKI

Interestingly, the serious problem in the AKI is also fibrosis. In 2016, Zou et al. demonstrated that in rats with unilateral IRI treated with hUC-MSC-EVs, decreased fibrosis was associated with increased angiogenesis. EVs promoted renal capillary formation by delivering to the tubular epithelial cells both vascular endothelial growth factor (VEGF) and RNA related to this process. Relieved fibrosis was evidenced by the reduction of the collagen deposition in damaged tissue and decreased expression of alpha-smooth muscle actin (α-SMA), protein expressed by myofibroblasts, known as a marker of the epithelial–mesenchymal transition process (EMT) [17].

A similar study was conducted by Ju et al. in 2015, where administration of hUC-MSC-MVs into the rats with ischemia–reperfusion injury increased hepatocyte growth factor (HGF) levels in TECs that was correlated with reduced renal tubular fibrosis [67].

Microscopic images revealed by Zou et al. in the group of rats treated with hUC-MSC-MVs showed the decrease in collagen deposition in the damaged kidney linked with decreased level of α-SMA. Interestingly, these changes were not accompanied by a change in TGF-β (tumor growth factor β) expression that may indicate that MVs enhancing the tissue repair by increasing the level of HGF (hepatocyte growth factor).

As it was shown, the treatment with hWJMSC-MVs decreases the expression of CX3CL1, and contributes to the reduction of inflammation in rats with IRI by reduction of interstitial fibrosis, that is evidenced by reduced collagen accumulation and decreased expression of α-SMA in kidney tissues [17].

Interestingly, EVs isolated from other populations of mesenchymal stem cells were also able to counteract fibrosis, for example, Lin et al. in 2016 indicated that providing aMSC-EXOs to rats with bilateral IRI also attenuated fibrosis. This was confirmed by the reduced levels of profibrotic proteins—p-Smad3 (phosphorylated mothers against decapentaplegic homolog 3) and TGF-β. At the same time, an increased expression of p-Smad1/5 and BMP-2 (bone morphogenetic protein 2) counteracting in this process was observed [68].

This anti-fibrotic effect was also confirmed by Lindoso et al. in 2014, who showed this effect after hBMSC-EVs application into the in vitro obtained IRI model in mice [52].

The protective effect of hBMSC-EVs was mediated by miRNAs, which regulates SMAD4. The protein encoded by it gene plays a significant role in EMT initiated by TGF-β1. Authors showed that the expression of SMAD4 in PTECs was reduced after incubation with EVs, that confirmed, that miRNAs could regulate the function of EVs [52].

In addition, Zhang et al. in 2020 reported that intravenous administration of hUC-MSC-EVs to mice with IRI reduced fibrosis by inhibiting the expression of Snail, a gene encoding a zinc finger transcription factor known to trigger EMT [69].

Interestingly, EVs overexpressing oct-4 (octamer-binding transcription factor 4) further reduced Snail expression, and thus inhibited fibrosis more effectively.

## Future perspectives

It is worth to note that certain cancers like leukemias, lymphomas, multiple myelomas (MM) and renal cell cancer (RCC) in both human and veterinary patients are related with the higher risk of AKI development, not only due to the accompanying kidney involvement, but also due to the hypotension, sepsis, administration of different anti-fungal and anti-bacterial agents, cytotoxic or immunosuppressive drugs and even hematopoietic stem cell transplantation (HSCT) and tumor lysis syndrome (TLS). The appropriate scheme of cancer treatment seems to be an ideal preventive strategy from AKI development [70].

Actual treatment strategies in AKI involve not specific procedures and have many disadvantages and complications, e.g., the most used fluid therapy should be carefully monitored due to its impact on the increased morbidity and mortality in both human and animals.

Searching for the new advanced and target AKI treatment strategy included also using a MSC cells that seems to be a promising tool in such situation; however, the lack of knowledge about their molecular mechanism of action during the regeneration processes in AKI seems to be a main cause that limits their use in the clinical practice.

However, from the other side, to undoubted advantages of using MSC-based therapies belong the fact that MSCs could be easily isolated from various sources including peripheral blood, bone marrow, adipose tissue, umbilical cord blood, amniotic membrane and fluids, placenta and even breast milk or dental pulp and etc. Moreover, not without significance are the facts that MSCs have the ability to differentiate into the various cell types, beneficial immunologic properties and are relative ease to culture in in vitro conditions [71].

Currently, such type of therapy is used primarily, in treating many degenerative diseases (osteoporotic in joints) and in the regenerative medicine of bones and cartilages. Its potential benefits are reflected also in the plastic surgery, aesthetic medicine and in treatment of cardiovascular, nervous and endocrine disorders. Furthermore, their application in the cell transplantation has been popular for years [72].

The main problem using stem cell-based therapies is the risk of tumorigenesis due to the MSCs ability to proliferate, its high viability and strong resistance to the apoptosis. Moreover, the long-term process of their culturing in in vitro conditions required to achieve the appropriate amount and properties of MSCs could cause genetic and chromosomal aberrations, that can lead to development of tumors in the recipient.

In AKI treatment, MSCs-based therapy has the protective potential, although conducted studies showed that injected MSCs mostly incorporate into the tubules, vessels and other compartments of kidneys due to their subcellular mechanism of action that handicaps their protective function. As it was proven, their protective potential in AKI treatment seems to be a result of released by them EVs [16].

The growing interest of EVs-derived from MSCs in AKI treatment is, on the one hand, the result of the lack of target, specific therapy, from the other is a result of the observation, that EVs obtained from MSCs have a potential to modulate anti-inflammatory response and have a significant impact on the cell apoptosis/necrosis, oxidative stress, anti-fibrotic and even on the proangiogenic processes, that are all included in the process of the tissue regeneration.

Encouraging in this case are the results of previously performed preclinical studies that confirmed the role of EVs-derived from MSCs in the improving of kidney conditions regardless of the cause of the main disease. The preliminary results with using EVs in the therapy of kidney injuries indicating their positive impact on the renal function reflected by the ratio of GFR and encouraging to continue these studies [22, 41].

Moreover, the low immunogenicity and high biocompatibility as well as the ease to penetrate the barriers make them an ideal tools for further AKI therapies in both human and animals.

The problem is that still the lack of the optimal protocols allows on the appropriate isolation and storage of EVs obtained from different sources of MSCs. At the same time, the problem is how to obtain the identical effectiveness of different batches of EVs [73].

In summary, EVs derived from MSCs seem to be a good alternative tool in the AKI treatment, due to their key role in the regulation of processes associated with the proper kidney function, although their heterogeneity could limit the beneficial effects.

The main problems, whose need to be solved before using EVs released from MSCs in the AKI treatment, are the determination of its safety, assessment of the amounts guaranteeing the expected protective effect, their pharmacodynamics, pharmacokinetics and accurate biodistribution allowing to choose an appropriate administration route [73].

Nevertheless, the beneficial properties of EVs derived from MSCs cells in AKI allow to suppose that therapy based on the EVs fills the gap between the classical and stem cell-based therapies in AKI in the near future.

## Data Availability

Not applicable.

## Abbreviations

AKI: Acute kidney injury
AKT: Protein kinase
aMSCs: Adipocyte-derived mesenchymal stem cells
ApoBDs: Apoptotic bodies
ARE: Anti-oxidant response element
ARF: Acute kidney failure
αSMA: α-Smooth muscle actin
Bax: Bcl-2 associated X
Bcl-2: B-cell CLL/lymphoma 2
BM: Bone marrow
BMP2: Bone morphogenetic protein 2
BUN: Blood urea nitrogen
Casp: Caspase
CCR-2: C-C motif chemokine receptor 2
CKD: Chronic kidney disease
CMJ: Corticomedullary junction
ConA: Concanavalin A
COX-2: Cyclooxygenase
Cr: Creatinine
DAMPs: Damage-associated molecular patterns
DCs: Dendritic cells
DRP1: Dynamin-related protein 1
EMT: Epithelial–mesenchymal transition process
ERK: Extracellular signal regulated kinase
EXOs: Exosomes
EVs: Extracellular vesicles
GFR: Glomerular filtration rate
GvHD: Graft versus host disease
HGF: Hepatocyte growth factor
HO-1: Heme oxygenase01
HOCl: Hypochlorous acid
H_2_O_2_: Hydrogen peroxidase
HSP: Heat shock protein
hUC-MSC: Human umbilical cord mesenchymal stem cells
hWJMSCs: Human stem cells derived from Wharton’s jelly
ICAM: Intracellular adhesion molecule
IDO: Indoleamine 2,3-dioxygenase
IFNγ: Interferon gamma
IL: Interleukin
ILVs: Intraluminal vesicles
iNOS: Inducible nitric oxide synthase
IRI: Ischemic reperfusion injury
IRIS: International Renal Interest Society
LAMs: Large animal models
MAF: Macrophage migration inhibitor
MCP-1: Monocyte chemoattractant protein 1
MDA: Malondialdehyde
MM: Multiple myeloma
MPs: Microparticles
MSCs: Mesenchymal stem cells
MVs: Microvesicles
MVBs: Mutivesicular bodies
NFκβ: Nuclear factor kβ
NK: Natural killers
NO: Nitric oxide
Nrf2: Nuclear factor erythroid-2 (related factor 2)
NRLP3: NLR family pyrin domain containing 3
NSAID: Non-steroidal anti-inflammatory drugs
O_2_: Superoxide anion
Oct-4: Octamer binding transcription factor 4
OH: Hydroxyl radical
OS: Oxidative stress
PAI-1: Plasminogen activator inhibitor I
PBMCs: Peripheral blood mononuclear cells
PD-L1: Programmed death ligand-1
PEG: Polyethylene glycol
pFUS: Pulsed focused ultrasound
PGE2: Prostaglandin E2
PI-3K: Phosphatidylinositol 3-kinase
p-SMAD3: Phosphorylated mothers against decapentaplegic homolog 3
PTEC: Proximal tubular epithelial cells
RCC: Renal cell cancer
RBF: Reduced renal blood flow
RNS: Reactive nitrogen species
ROS: Reactive oxygen species
RRT: Renal replacement therapy
RTEC: Renal tubular epithelial cells
Sema3A: Semaphorin 3A
SNARE: SNAP receptor
SOD: Superoxide dismutase
TGFβ: Tumor growth factor β
TLR: Toll-like receptors
TNFα: Tumor necrosis factor α
TnT: Nanotubes
VDA: Vitamin D analog
VEGF: Vascular endothelial growth factor
